# Role of Inhaled Steroids in Vascular Airway Remodelling in Asthma and COPD

**DOI:** 10.1155/2012/397693

**Published:** 2012-10-11

**Authors:** Alfredo Chetta, Dario Olivieri

**Affiliations:** Department of Clinical and Experimental Medicine, Respiratory Disease and Lung Function Unit, University of Parma, Padiglione Rasori, Azienda Ospedaliero-Universitaria, Viale Rasori 10, 43125 Parma, Italy

## Abstract

In chronic obstructive airway diseases, such as asthma and chronic obstructive pulmonary disease (COPD), changes in bronchial microvasculature are present in response to inflammatory stimuli. Vascular changes may significantly contribute to airway wall remodelling. Angiogenesis and vascular leakage are prevalent in asthma, while vasodilation and vascular leakage dominate in COPD. An endothelial dysfunction may be present both in asthma and in COPD. Vascular changes may occur simultaneously with the thickening of the airway wall and the narrowing of the bronchial lumen. Consequently, pharmacological control of bronchial vascular remodelling may be crucial for symptom control in asthma and COPD. In asthmatic airways, inhaled steroids can downregulate vascular remodelling by acting on proangiogenic factors. Additionally, studies on combination therapy with long-acting **β**2-agonists and inhaled steroids have provided evidence of a possible synergistic action on components of vascular remodelling in asthma. In COPD, there is less experimental evidence on the effect of inhaled steroids on airway microvascular changes. Importantly, vascular endothelial growth factor (VEGF), the most specific growth factor for vascular endothelium, is crucially involved in the pathophysiology of airway vascular remodelling, both in asthma and COPD. The inhibition of VEGF and its receptor may be useful in the treatment of the vascular changes in the airway wall.

## 1. Introduction

In chronic obstructive airway diseases, such as asthma and chronic obstructive pulmonary disease (COPD), changes in bronchial microvasculature are present in response to inflammatory stimuli [1–4]. Vascular changes may significantly contribute to airway wall remodelling in asthma and COPD [1–4]. Importantly, changes in the microvasculature may occur simultaneously with the thickening of the airway wall, thereby causing a narrowing of the bronchial lumen and a decrease in the airway patency [3, 5]. Consequently, pharmacological control of bronchial vascular remodelling may be crucial for symptom control in asthma and COPD. Among the drugs commonly used to treat chronic obstructive airway diseases, inhaled steroids are the most effective on the inflammatory process in bronchial airways [1, 4].

This overview describes the features of the vascular component of airway remodelling in asthma and COPD and its possible functional consequences. Moreover, we focus on the effects of inhaled steroids on vascular remodelling in asthma and COPD.

## 2. Vascular Remodelling in Asthma

**Figure 1:**
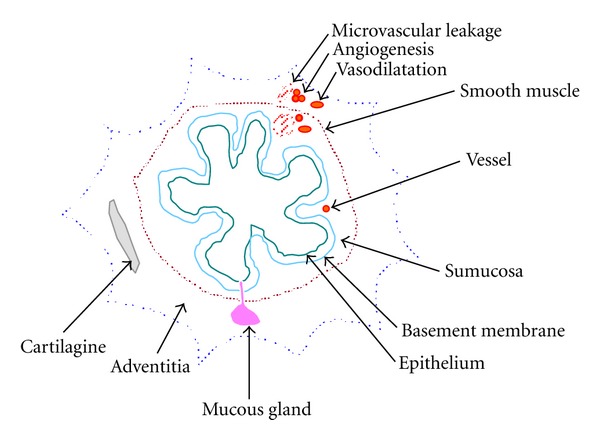
Schematic representation of bronchial airways wall. In asthma and COPD changes in bronchial microvasculature, such as angiogenesis, vasodilatation, and microvascular leakage, are present in response to inflammatory stimuli. Considering conventional therapy, only corticosteroids can positively act on all aspects of vascular remodelling.

Airway wall remodelling is a distinctive feature of bronchial asthma [6]. In asthmatic airways, structural changes occur in all three layers of the airway wall: the inner wall, the outer wall, and the smooth-muscle layer (Figure 1). Basically, the remodelling consists in the shedding of the epithelium, the increase in mucous glands, thickening of the reticular basement membrane, hypertrophy and hyperplasia of airway smooth muscle, and in qualitative and quantitative changes in airway blood vessels [7].

The vascular component of airway remodelling in asthma is arousing more and more interest in researchers, because bronchial microvasculature has several functions that are essential for maintaining homeostasis. These functions include provision of oxygen and nutrients, temperature regulation, and humidification of inspired air, as well as being the primary portal of the immune response to inspired organisms and antigens [8]. Although there may be methodological problems in measuring the vascular area of the bronchial mucosa [9], however, there is evidence that structural vascular changes may significantly occur in the mucosa of the asthmatic airways. In bronchial asthma, airway microvascularity may change by increasing vessel calibre through vasodilation, angiogenesis, and by inducing interstitial edema within the airway wall, as a consequence of microvascular leakage [1–4]. Furthermore, in asthmatic patients the airway microvascularity changes are associated with an increase in airway blood flow (*Q*_aw_) and blunted *β*_2_-adrenergic vasodilator responsiveness [10], the latter finding suggesting the presence of endothelial dysfunction. The role of endothelial dysfunction in the pathogenesis of bronchial asthma is not yet fully clarified and it still remains to ascertain whether or not it is an epiphenomenon [11]. 

In asthmatic airways, a link between airway inflammation and changes in microvascularity has been suggested [12, 13]. Many mediators and growth factors which are directly related to airway inflammation in asthma, such as histamine, the major preformed mast cell mediator, prostaglandins, leukotrienes, and cytokines, can simultaneously induce vascular responses, such as angiogenesis vasodilation and microvascular leakage [14–16]. However, the interpretation of the specific role of many of these factors is often indirect since it is based on studies of animal models, *in vitro* studies, or studies performed on sites other than asthmatic airways.

 If only *in vivo* and asthmatic airway studies are considered, vascular endothelial growth factor (VEGF) seems to be particularly involved in the vascular changes in asthma. VEGF is a potent multifunctional cytokine with several important effects such as acting on angiogenic sprouting as well as on vascular leakage and permeability [17]. In an *in vivo *study by Hoshino and coworkers [18], the levels of three angiogenic factors, VEGF, basic fibroblast growth factor (bFGF), and angiogenin were significantly increased in the airways of asthmatic subjects compared to controls. Moreover, the authors found a significant correlation between microvascularity and the amount of angiogenic factors. Another interesting result of this study was that in the submucosa the cells positive to angiogenic factors were CD34+, eosinophils, and macrophages [18]. 

The results of the Hoshino study were subsequently confirmed and expanded on in our lab. In patients with mild-to-moderate asthma, we found that in biopsy specimens of bronchial mucosa the expression of VEGF was upregulated as compared to controls [19]. We also found that VEGF was related to the number of vessels and to the thickness of the basement membrane [19]. Previous *in vitro *studies [20, 21] have shown that VEGF expression can be associated with fibrosis and the remodelling processes. Interestingly, we have shown a relationship between VEGF and the number of mast cells [19]. Thanks to colocalization analysis we were able to demonstrate that in asthmatics, mast cells were a main cellular source of VEGF. Our findings further support the view that mast cells can release VEGF and contribute to the angiogenesis process in asthma. 

Notably, according to the protease content, mast cells are a heterogeneous group of cells and can be divided into those that express tryptase but not chymase and those that express tryptase and chymase. In asthmatic airways, we have demonstrated that a substantial subset of mast cells (about 25%) express chymase [22]. We also have found that there is a close relationship between VEGF expression and chymase positive mast cells [22]. Colocalization analysis on sequential sections of bronchial mucosa showed chymase positive mast cells as a source of VEGF [22]. Thus, our results suggest that chymase positive mast cells may play a role in the vascular component of airway remodelling in asthma.

The remodelling process might be beneficial in airway disease and it has been suggested that airway wall-thickening protects against airway narrowing and air trapping by making the airway wall stiffer [23]. However, the detrimental effects seem to be more important than the protective ones. Notably, remodelling may increase the irreversible component of airway obstruction, accelerate the decline in pulmonary function, and facilitate the persistence of airway hyperresponsiveness, the loss of smooth muscle stretch relaxation, the increase in contractile response, and the loss of elastic recoil [24]. The clinical relevance of the various airway structural changes, particularly of airway vascular remodelling, remains to be determined. 

There are very few results concerning the impact of airway microvascularity on clinical features and pulmonary function in asthma. Some studies [3, 25, 26] showed a direct relationship between an increase in microvascularity, expressed as the density of vessels, and the severity of asthma. Thus, the airway wall of severe asthmatics was more vascularized than that of mild-to-moderate patients. In the study by Orsida et al. [27], which included patients both having and not having steroid treatment, the density of vessels was found to be weakly related to bronchial hyperresponsiveness to methacholine, showing that vessel density was greater in those patients with the most marked hyperresponsiveness. The same study also showed a significant positive correlation between percentage change in forced expiratory volume in the 1st second (FEV_1_) after bronchodilator and number of vessels/mm^2^ for the asthma patients overall. Taken together the findings of the Orsida et al. study [27] suggest that the increased microvascularity in airways may have pathophysiological relevance in asthma. 

Hashimoto et al. [3] assessed the relative impact of the increased microvascularity of medium and small airways on airflow limitation in nine asthmatic patients, who underwent lobectomy or pneumonectomy for a solitary peripheral carcinoma. In this study, the FEV_1_, expressed as a percentage of predicted value, correlated with the vascularity in the inner area of the medium airways, but not in the small airways. Thus, though correlation does not imply causality, the results of the study by Hashimoto et al. [3] suggest that the enhanced vascularity in the inner area of the medium airways, but not in the small airways, might contribute to airflow limitation in patients with asthma. 

## 3. Vascular Remodelling in COPD

Epithelial damage and mucus gland hyperplasia are the structural changes which mostly characterize airway wall-remodelling in COPD, even if subepithelial collagen deposition, increased smooth muscle, and vascular changes can be also observed [24] (Figure 1). When the structural changes of the airway wall in asthma and COPD are compared, the vascular component of airway remodelling seems to be less represented in COPD [24]. Thus, in contrast to asthma, angiogenesis in the airway wall does not appear to be a typical feature of COPD.

Up to now, *in vivo* studies concerning vascular changes of the bronchial wall in COPD airways have provided different results. Unlike the study by Kuwano et al. [28], which did not find any significant difference in vascular area between patients with mild COPD and controls, more recent studies by Hashimoto et al. [3], Calabrese et al. [29], and Zanini et al. [4] have shown that the vascular area of the airway wall was increased in COPD patients, as compared to controls. Notably, Hashimoto et al. [3] showed that, compared to controls, the vascular area was increased in the small airways, but not in the medium airways. Yet, in the same study no correlation was found between the degree of vascularity and airflow limitation in patients with COPD. 

In the study by Calabrese et al. [29], the authors examined mucosal microvascularity in large airways of current smokers with COPD and they found a significant difference in vascular area in comparison to controls. Similarly to the Hashimoto et al. study [3], in this study [29] no correlation between microvascularity data and clinical and functional data were observed. No difference was also found between symptomatic smokers with normal function and symptomatic smokers with moderate COPD. Finally, the authors showed an increase in bronchial vascularity, which was associated with higher cellular expression of VEGF and vascular expression of *α*v*β*3 integrin [29]. *α*v*β*3 integrin is an adhesion molecule that is upregulated in new vessel proliferation in response to angiogenic stimuli, while it is expressed at low levels or absent on resting endothelium [30]. Interestingly, it has been recently observed that VEGF and *α*v*β*3 integrin can synergistically exert angiogenic effects [31].

Zanini et al. [4] assessed bronchial vascular remodelling in the central airways of COPD patients and showed that the bronchial vascular area and vessel size were increased, as compared to those of control subjects. Moreover, they found a higher bronchial expression of VEGF, bFGF, and transforming growth factor-beta (TGF-*β*) in COPD patients, as compared to controls, and provided the evidence that the vascular component of airway remodelling was positively related to the bronchial expression of VEGF and TGF-*β*.

To further confirm the role of vascular changes in the airway walls of COPD patients, Soltani et al. [32] have recently shown that airway remodelling in smokers and in patients with mild-to-moderate COPD was associated with fragmentation of the reticular basement membrane and altered distribution of vessels in the airway wall. The reticular basement membrane showed fragmentation and splitting in both the current smokers group and the ex-smoker with COPD group compared with healthy nonsmokers. Reticular basement membrane fragmentation was also equally present in ex-smokers with COPD. The number of vessels staining for VEGF in the reticular basement membrane was higher in both the current smoker and ex-smoker COPD groups, as compared to healthy nonsmokers. These morphological features may have potential physiological consequences in COPD. In that study [32], the authors found that in current smokers with COPD, VEGF vessel staining positively correlated with FEV_1_, expressed as percent of predicted value.

Similarly to what was found in asthmatic airways, VEGF also seems to be a protein crucially involved in the vascular remodelling of COPD. In the study by Kranenburg and coworkers [33], COPD airways were associated with an increased expression of VEGF in several cell populations, such as in the bronchial, bronchiolar, and alveolar epithelium and in bronchiolar macrophages, as well as airway smooth muscle and vascular smooth muscle cells in both the bronchiolar and alveolar regions. These authors also found a significant inverse correlation between VEGF and FEV_1_ in bronchial mucosal microvessels and airway smooth muscle cells, bronchiolar epithelium, and medial vascular smooth muscle cells of larger pulmonary arteries associated with bronchiolar airways. Although association does not establish causality, taken together, these findings strongly suggest a role for VEGF in airway and vascular remodelling, and thereby in the development of airway obstruction in COPD.

Vascular structural changes in airway mucosa of COPD patients do not seem to imply any quantitative change in *Q*_aw_, even if qualitative changes may occur. Contrary to what has been shown in asthmatic patients in whom *Q*_aw_ levels were increased [10], patients with COPD may show normal *Q*_aw_ levels. *Q*_aw_ was measured in age-matched healthy current smokers, healthy ex-smokers, ex-smokers with COPD, and healthy lifetime nonsmokers and it did not significantly differ among groups [34]. The authors also found that Salbutamol inhalation increased *Q*_aw_ significantly in lifetime nonsmokers and healthy ex-smokers, but not in healthy current smokers and ex-smokers with COPD [34]. Similarly, Paredi et al. [35] found that *Q*_aw_ was similar in patients with COPD, as compared to healthy subjects and that, after the inhalation of 200 mcg of Salbutamol, it was significantly more elevated in controls in comparison to COPD patients. Taken together the findings of these studies [34, 35] indicate that cigarette smoking as well as COPD are associated with a blunted vasodilator response to inhaled Salbutamol in the airway, as an expression of endothelial dysfunction.

## 4. Inhaled Steroids and Vascular Remodelling in Asthma and in COPD

 Steroids can positively affect all three main aspects of the vascular component of airway remodelling in asthma: vasodilatation, increased vascular permeability, and angiogenesis [36]. There is evidence that inhaled fluticasone propionate can act on the blood flow in the bronchial mucosa by causing vasoconstriction [37]. In addition, the vasoconstrictor response was greater in subjects with higher baseline airway mucosal blood flow irrespective of whether they were asthmatic or normal [37]. These findings suggest that inhaled steroids have potentially beneficial effects in asthma, that are not related to their anti-inflammatory action.

Steroid-induced vasoconstriction is complex and different cellular mechanisms may occur [38]. These mechanisms are too rapid to involve gene expression and have termed nongenomic actions [38]. Rapid nongenomic mechanisms of corticosteroid actions are initiated by specific interactions with membrane-bound or cytosolic glucocorticoid receptors or nonspecific interactions with the cell membrane [38]. It is of note that steroids can act via noradrenergic neurotransmission by potentiating the physiological effect of noradrenaline. It has been shown that corticosteroids can increase the sensitivity of vascular smooth muscle to the vasoconstrictor effects of noradrenaline, an effect reversed by infusions of arachidonic acid and prostacyclin [39]. Steroids can also exert a vasoconstrictive action by nonadrenergic neurotransmission mechanisms and/or by acting on nitric oxide levels [40, 41]. 

Steroids can act not only on airway blood flow, but also on endothelial dysfunction and airway blood vessel density of asthmatic airways. In steroid-naïve patients with asthma, who had a blunted vasodilator response to Salbutamol in the airways, inhaled steroids administered for several weeks have been found to restore normal *β*_2_-adrenergic responsiveness [42]. It has been also shown that high doses of inhaled beclomethasone dipropionate given for six months were able to reduce airway wall vascularity, evaluated as both vessel number and percent vascularity, in mild-to-moderate asthmatic patients [43]. Similarly, high doses of fluticasone propionate were able to decrease the number of vessels and percent of vascular area as well as VEGF expression in the bronchial mucosa of asthmatic patients [1]. Moreover, Asai et al. [44] found that VEGF levels in induced sputum of asthmatic patients were reduced after long-term treatment with beclomethasone dipropionate. Kanazawa et al. [45] also showed that fluticasone treatment was able to reduce VEGF and angiopoietin-1 levels and to improve airway microvascular permeability. Taken together these findings support the view that steroids can downregulate vascular remodelling in asthmatic airways by acting on proangiogenic factors. In addition, steroid effects on endothelial dysfunction and airway blood vessel density were likely to reflect a genomic action as they were observed several hours after the last steroid dose.

Studies on combination therapy with long-acting *β*_2_-agonists and inhaled steroids have provided some interesting results on a possible synergistic action on components of vascular remodelling in asthma. In asthmatic patients treated with low-dose inhaled steroids, there was a positive effect on the vascular component of airway remodelling after a-three-month treatment with inhaled salmeterol, with a significant decrease in the number of vessels [46]. A synergistic action was not observed between LTRAs and steroids. When montelukast was combined with fluticasone propionate in asthmatic patients, its effect in reducing airway mucosal blood flow was not greater than when it was administered alone [47]. 

Current treatment for COPD patients is predominantly based on corticosteroids and bronchodilators, such as *β*_2_-agonists and anticholinergics [48]. The effect of these drugs on bronchial microvascularity has been scarcely investigated in COPD airways. Recently, we have demonstrated that in COPD patients airway vascular remodelling may be affected by inhaled steroids [4]. As compared to untreated COPD patients, COPD patients treated with high doses of beclomethasone showed lower values of vascular area and VEGF and bFGF expression [4]. Interestingly, in ex-smokers with COPD, who showed a blunted vasodilator response to inhaled Salbutamol in the airways, combined glucocorticoid/long-acting *β*_2_-adrenergic agonist treatment restored the responsiveness to the *β*_2_-adrenergic agonist [34]. The role of endothelial dysfunction in the pathophysiology of COPD and the clinical implications of its reversibility induced by inhaled steroids are still subject of speculation [11]. However, it is conceivable that inhaled steroids have a beneficial effect even in extrapulmonary vascular dysfunction and this fact could explain the reduction in the incidence of fatal and nonfatal cardiovascular events associated to steroid treatment in COPD patients [11]. 

## 5. Conclusions

In conclusion, there is evidence that the airway wall is more vascularized in asthmatic and COPD patients than in healthy subjects, though this phenomenon is more evident in asthma than in COPD. Notably, angiogenesis and vascular leakage seem to be prevalent in asthma, while vasodilation and vascular leakage seem to prevail in COPD. Although airway mucosal vessels can contribute to airway flow obstruction by angiogenetic processes, vasodilation, and increased microvascular permeability, the clinical and functional relevance of vascular remodelling remain to be determined in chronic obstructive airway diseases.

Considering conventional antiasthma therapy, only corticosteroids can positively act on the three aspects of vascular remodelling: angiogenesis, dilation, and permeability, and there is evidence that steroids can downregulate vascular remodelling in asthmatic airways by acting on proangiogenic factors. It is likely that corticosteroids may affect airway microvascularity changes also in COPD. Interestingly, inhaled corticosteroids may positively act on endothelial dysfunction both in asthma and COPD. VEGF is the most specific growth factor for vascular endothelium and it is crucially involved in the pathophysiology of airway vascular remodelling, both in asthma and COPD. The inhibition of VEGF and its receptors may be potentially useful in the treatment of vascular changes in the airway wall.
